# The increase of alpha-melanocyte-stimulating hormone in the plasma of chronic fatigue syndrome patients

**DOI:** 10.1186/1471-2377-10-73

**Published:** 2010-08-23

**Authors:** Nobue Shishioh-Ikejima, Tokiko Ogawa, Kouzi Yamaguti, Yasuyoshi Watanabe, Hirohiko Kuratsune, Hiroshi Kiyama

**Affiliations:** 1 Department of Anatomy & Neurobiology, Osaka City University, Graduate School of Medicine, Osaka, 545-8585, Japan; 2 Department of Physiology, Osaka City University, Graduate School of Medicine, Osaka, 545-8585, Japan; 3 Center for Molecular Imaging Science, RIKEN, Kobe, Hyogo 650-0047, Japan; 4 Faculty of Health Science for Welfare, Kansai University of Welfare Sciences, Kashihara, Osaka, 582-0026, Japan; 5 Department of Comparative Pathophysiology, Graduate School of Agricultural & Life Sciences, The University of Tokyo, Bunkyo-ku, Tokyo, 113-8657, Japan

## Abstract

**Background:**

Despite extensive research, no reliable biological marker for chronic fatigue syndrome (CFS) has yet been identified. However, hyperactivation of melanotrophs in the pituitary gland and increased levels of plasma alpha-melanocyte-stimulating hormone (α-MSH) have recently been detected in an animal model of chronic stress. Because CFS is considered to be caused partly by chronic stress events, increased α-MSH plasma levels may also occur in CFS patients. We therefore examined α-MSH levels in CFS patients.

**Methods:**

Fifty-five CFS patients, who were previously diagnosed within 10 years of with the disease, were enrolled in this study. Thirty healthy volunteers were studied as controls. Fasting bloods samples were collected in the morning and evaluated for their plasma levels of α-MSH, adrenocorticotropic hormone (ACTH), serum cortisol and dehydroepiandrosterone sulfate (DHEA-S). Mean levels of α-MSH were compared between the CFS and control groups using Welch's *t *test.

**Results:**

The mean plasma α-MSH concentration in the CFS group (17.9 ± 1.0 pg/mL) was significantly higher than that in healthy controls (14.5 ± 1.0 pg/mL, p = 0.02). However, there was a wide range of values in the CFS group. The factors correlated with the plasma α-MSH values were analyzed using Spearman's rank correlation. A negative correlation was found between the duration of the CFS and the plasma α-MSH values (p = 0.04, r_s _= -0.28), but no correlations with ACTH, cortisol or DHEA-S levels were identified (p = 0.55, 0.26, 0.33, respectively). The CFS patients were divided into two groups: patients diagnosed for ≤ 5 years' duration, and those diagnosed for 5-10 years' duration. They were compared with the healthy controls using one-way ANOVA and Tukey-Kramer multiple comparison tests. The mean α-MSH concentration in the ≤ 5 years group was 20.8 ± 1.2 pg/mL, which was significantly higher than that in the healthy controls (p < 0.01). There was no significant difference between the 5-10 year group (15.6 ± 1.4 pg/mL) and the healthy controls.

**Conclusions:**

CFS patients with a disease duration of ≤ 5 years had significantly higher levels of α-MSH in their peripheral blood. α-MSH could be a potent biological marker for the diagnosis of CFS, at least during the first 5 years after onset of the disease.

## Background

According to the guidelines of the United States Centers for Disease Control and Prevention [1], chronic fatigue syndrome (CFS) is defined as persistent fatigue, not substantially relieved by rest, and accompanied by other specific symptoms for a minimum of 6 months. However, the etiology and pathophysiology of CFS remain unclear. Homeostatic systems are assumed to be impaired in CFS patients, leading to prolonged illness and chronic fatigue symptoms [2,3]. Several lines of study have addressed the possible causes of CFS. Psychological disorders such as depression, viral infections, autoimmune diseases, and prolonged stresses have all been considered as potential candidates [4-6], although the mechanisms by which these conditions cause the symptoms of CFS are still unclear. CFS is a highly heterogeneous and partly subjective illness, and no standard laboratory test is currently available for the reliable diagnosis of CFS. These heterogeneity and lack of reliable biomarkers result in lack of clues for exploring CFS etiology and pathology. To prevent this vicious circle, identifications of multiple objective biomarkers for CFS have thus long been sought. In this regard, the present study was aimed to establish a new biomarker for CSF.

Animal models of fatigue were established by Tanaka, and this model showed a decrease ability to exercise [7]. In this model, we recently found that prolonged stress caused various alterations in the brain and subsequent changes in the endocrine organs and the brain [8,9]. The most notable changes occurred in the pituitary gland. Prolonged stress resulted in overactivation of the melanotrophs in the pituitary gland and their eventual cell death. This overactivation and subsequent cell death were caused by altered dopamine expression in a specific region of the hypothalamus, suggesting that molecular alterations in the brain can lead to the dysfunction and further death of pituitary cells [9]. Significant increases in plasma α-MSH levels were observed in this animal model, and the removal of the pituitary gland totally suppressed the increase induced by the stimulus. These results suggested that hyper-activation of melanotrophs induced the over-secretion of α-MSH from the pituitary gland in response to continuous stress. We therefore hypothesized that a similar increase in plasma α-MSH levels reflecting the activation of melanotrophs under chronic stress might occur in humans, especially in patients suffering from persistent fatigue. In this study, we therefore compared plasma α-MSH levels in CFS patients with healthy controls during the first 10 years of the disease.

## Methods

### Study subjects

The study subjects included 55 patients with CFS (35.4 ± 1.1 years old) and 30 age-sex-matched healthy controls who did not complain of feeling fatigued and had no illness, as healthy controls (36.1 ± 1.6 years old). The CFS patients were diagnosed using the clinical criteria proposed by Fukuda (1994) [1] and were treated at the Osaka City University Hospital. Written informed consent was obtained from each patient prior to the study. The study was approved by the Ethical Committee of Osaka City University.

### Study protocol and methods

Venous blood was drawn between 9 and 10 am., after fasting since the previous day. Following centrifugation, plasma and serum fractions were frozen at -80°C until assay. Plasma α-MSH levels were measured using a commercial radioimmunoassay kit (Eurodiagnostica, Malmö, Sweden). The minimum detectable concentration of α-MSH was 3.9 pg/mL and the intra- and inter-assay coefficients of variation were 2.3% and 4.5%, respectively. The cross-reactivity with other proopiomelanocortin peptides (adrenocorticotropic hormone (ACTH) 1-24, ACTH 1-39, α-MSH and γ-MSH) was < 0.002%. Previous tests have shown that α-MSH concentrations in plasma are stable over long periods when properly stored at -80°C [10]. ACTH levels in plasma, and cortisol and dehydroepiandrosterone sulfate (DHEA-S) in serum were measured by the SRL Corp [11,12]. The durations of CFS were determined as the months from the first symptoms to the blood sampling day. We excluded patients who had been suffering from CFS for more than 10 years.

### Statistical analysis

Results are expressed as means ± standard error of mean (SEM). The differences between the CFS and healthy groups were determined using Welch's *t*-test. Correlations were analyzed using Spearman's rank test. Correlation coefficients were obtained using Microsoft Excel with the add-in software Statcel2 (Microsoft Co. Japan). Differences among healthy controls, and patients with shorter and longer durations of CFS were determined using Tukey's test following one-way ANOVA. p < 0.05 was considered statistically significant.

## Results

**Figure 1:**
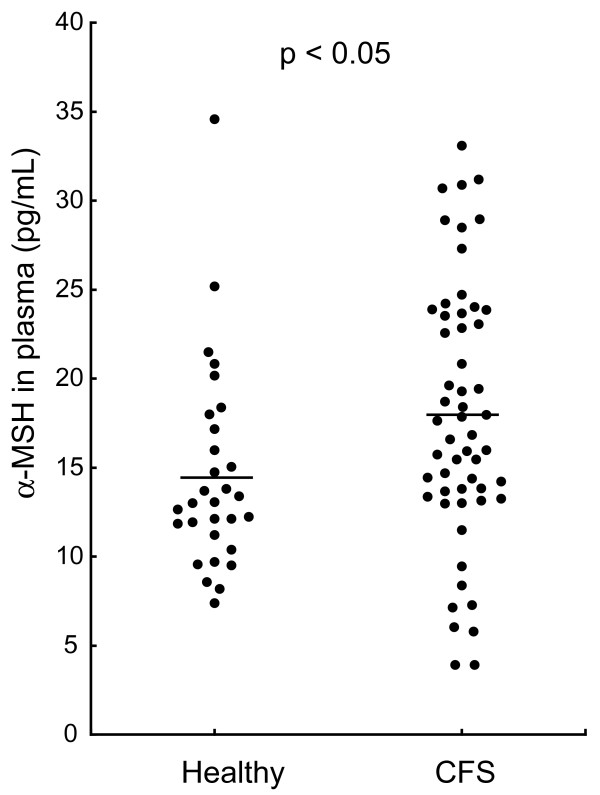
**Concentrations of α-MSH in plasma of healthy control and CFS patients**. α-MSH levels in CFS patients were significantly higher than in healthy controls (p < 0.05). The range of values in the CFS group was wider than that in the control group.

**Figure 2:**
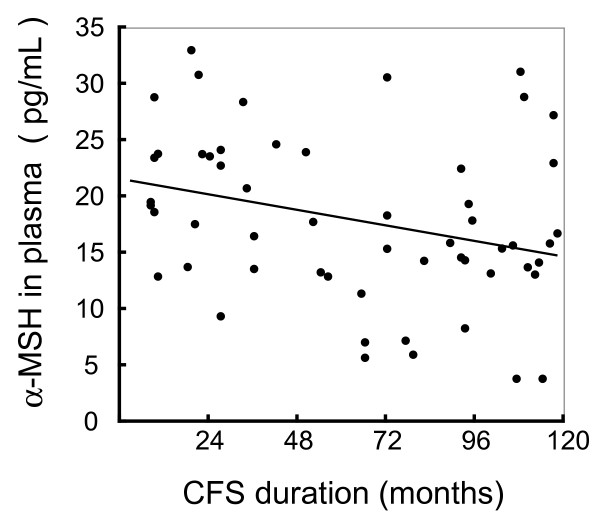
**Correlations between α-MSH concentrations and duration of CFS**. A significant correlation was observed between α-MSH and duration of the disease in CFS patients (*r *= -0.27, p < 0.05).

**Figure 3:**
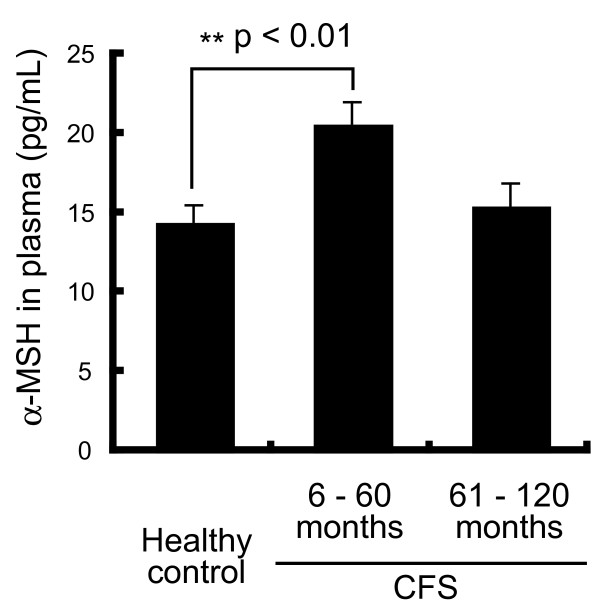
**Concentrations of α-MSH in CFS patients divided according to duration of CFS and in healthy controls**. The mean α-MSH level in the 6-60-month group was significantly higher than the levels in the other groups (p < 0.01 compared with the healthy group, p < 0.05 compared with the 61-120-month group). Values are given as means ± SEM.

The characteristics of the subjects are summarized in Table 1. The mean plasma concentration of α-MSH in the CFS group was 17.9 ± 1.0 pg/mL, which was higher than that in the healthy group (14.5 ± 1.0 pg/mL, p = 0.02). The variation in levels was greater in the CFS group than in the healthy group, as shown in Figure 1. We therefore investigated the factors correlated with α-MSH levels in the CFS group. There was a negative correlation between plasma α-MSH levels and months of CFS morbid period (with *r *= -0.28, p = 0.04, Figure 2 and Table 2). There were no correlations between α-MSH and the following: gender, age, visual analog scale, performance status score, body mass index (BMI), DHEA-S levels, ACTH levels, cortisol levels, blood pressure, prescribed medicine, or physical or mental symptoms [11-15]. In particular no significant difference in α-MSH levels between CFS patients with and without depression was observed (p = 0.11 *t*-test). We investigated the correlations between duration of CFS and levels of other stress-responsive hormones. There were no significant correlations between duration of CFS and plasma ACTH, or serum cortisol or DHEA-S (p = 0.55, p = 0.26 and p = 0.33, respectively) (Table 2). CFS patients were divided into two groups: a shorter CFS duration group (from 6-60 months) and a longer CFS duration group (from 61-120 months). As shown in Figure 3, the shorter duration group had significantly higher levels of α-MSH than the healthy controls (p = 0.002, by one-way ANOVA; p < 0.01 by Tukey's test). The characteristics of the two CFS groups are shown in Table 3, which shows that there were no significant differences in levels of other stress hormones between the shorter and longer duration groups. In addition we examined the differences of the onset symptoms among patients, either the sudden or gradual, and found no significant differences between.

**Table 1:** Characteristics of the subjects

	CFS	Healthy
	(n = 55)	(n = 30)
Age (y)	35.4 ± 1.1	36.1 ± 1.6
Male	20	11
Female	35	19
α-MSH (pg/mL)	17.9 ± 1.0	14.5 ± 1.0*

Results are given as mean ± SEM.

*p < 0.05 between CFS and healthy controls (Welch's *t*-test).

**Table 2:** Spearman's rank correlation analysis of stress hormones and CFS duration (months)

	n	*r*	p-value
α-MSH	55	-0.28	0.04*
ACTH	43	-0.10	0.55
Cortisol	43	-0.17	0.26
DHEA-S	43	0.15	0.33

*p < 0.05

**Table 3:** Characteristics of the subjects divided according to CFS duration

	6-60 months	61-120 months
Age	33.6 ± 1.7 (n = 25)	36.9 ± 1.5 (n = 30)
Male	9	15
Female	16	15
BMI (kg/m^2^)	21.4 ± 0.63 (n = 25)	21.1 ± 0.59 (n = 30)
α-MSH (pg/mL)	20.8 ± 1.2 (n = 25)	15.6 ± 1.4 (n = 30)*
ACTH (pg/mL)	29.3 ± 3.7 (n = 18)	23.44 ± 3.3 (n = 25)
Cortisol (mg/dL)	16.4 ± 1.6 (n = 18)	12.9 ± 1.2 (n = 25)
DHEA-S (mg/dL)	163.4 ± 16.5 (n = 18)	167.88 ± 15.7 (n = 25)

Results are given as mean ± SEM.

*p < 0.05 between 6-60-month group and 61-120-month group (Tukey's test).

## Discussion

In this study, we demonstrated that plasma α-MSH levels in CFS patients were significantly higher than those in normal healthy controls, and that there was a significant negative correlation between α-MSH concentrations and the duration of CFS. These results suggest that α-MSH could be a biomarker for CFS in patients who have suffered from the disease for less than 5 years. In contrast, we were unable to identify any significant correlation between the CFS morbid period and levels of ACTH, cortisol or DHEA-S. This suggests that well known stress markers such as ACTH and cortisol are not suitable markers for CFS [11,16]. α-MSH also has the advantage that its plasma levels remain relatively stable, both in terms of daily variations [17], and in terms of seasonal variations [18]. Furthermore, α-MSH levels in healthy controls fall within a relatively narrow range (Figure 1), as shown by both the current and previous reports [17-19]. Thus, 〈-MSH would have an advantage as a biomarker to diagnose CFS in terms of stability, because the level of 〈-MSH was not affected by acute stress and rhythmicity.

Circulating α-MSH could originate from the pituitary gland and/or blood cells. The results of experiments using a continuous stress rat model suggested that the α-MSH was produced by the pituitary gland. Dopaminergic neurons located in A14 (the hypothalamic periventricular region) project their axons to the intermediate lobe and suppress melanotroph activity via dopamine production. In our previous study, reduced dopamine synthesis in these neurons elicited hyper-activation of melanotrophs. This was confirmed by the application of a dopamine agonist, which suppressed the secretion of α-MSH from the pituitary gland [9]. Furthermore, removal of the pituitary gland resulted in suppression of the stress-induced increase of the plasma α-MSH, suggesting that the chronic stress-induced increase of α-MSH originated from the pituitary gland. These results suggest that dopamine synthesis is suppressed in some hypothalamic neurons in CFS patients, and melanotrophs may thus be hyper-activated. Intriguingly, Sharpe et al. demonstrated an increase in prolactin response in CFS, and suggested the possibility that CFS patients could have an abnormal dopamine neurotransmission [20]. Overall, these results suggest that a disorder of the hypothalamic dopaminergic neurons or dopamine neurotransmission might occur in CFS patients, and that this could further affect pituitary hormone secretion. Although the structure of the intermediate lobe is less clear than that seen in rodents, melanotrophs are found in the intermediate area of the human pituitary gland. It thus seems likely that the increase in circulating α-MSH in CFS patients originates from the pituitary gland in response to persistent and prolonged stress. However, some human studies have reported that some blood cells in patients with sepsis and some inflammatory diseases secrete α-MSH [21], and the possibility that α-MSH is released by some blood cells in CFS patients following prolonged stimulation cannot be ruled out.

We found a negative correlation between α-MSH levels and the duration of CFS. As the duration increased, the α-MSH level fell to similar levels to that seen in the healthy controls. This may be a result of melanotroph dysfunction following prolonged stimulation. In a rat model, melanotrophs subjected to continuous stress for more than 5 days showed degenerative features due to hyper-secretion of α-MSH, and the raised α-MSH levels fell five days after stimulation [9]. Thus, melanotrophs in human with CFS are likely to become exhausted and impaired by prolonged stress. It is also possible that the melanotrophs become desensitized following prolonged stimulation, or that the prolonged high level of α-MSH may activate an unidentified feedback system from the periphery.

The functional significance of circulating α-MSH remains unclear, though α-MSH has been shown to have an anti-inflammatory function [17]. *In vitro*, lipopolysaccharide-stimulated inflammatory cytokines were suppressed by the application of α-MSH [22-24]. In accordance with this *in vitro *study, increases in plasma α-MSH have also been reported in some inflammation-associated diseases, such as HIV [10] and sepsis [21]. Intriguingly, elevated α-MSH levels were observed particularly in non-progressive HIV patients and in sepsis patients with lower plasma tumor necrosis factor-α (TNF-α) levels. These observations suggest that increased α-MSH levels may have suppressed the inflammatory responses and consequently inhibited the progression of HIV and the increase in TNF-α. Some CFS patients in the current study also had symptoms such as slight fever, pharyngalgia and lymphadenopathy, though no significant correlations between α-MSH levels and these symptoms were observed (data not shown). However in this study we could not conclude that the increase of α-MSH was for the anti-inflammatory function. In some literatures, increased levels of α-MSH have also been demonstrated in patients suffering from congestive heart failure (CHF) [19] and obesity [14,15]. None of the patients in the current study had CHF, and eight had BMIs of > 25. We were thus unable to address the possible correlation between CFS and CHF, and no correlation between α-MSH levels and BMI was found in the patients examined. Consequently it is hard to explain some functional significance in the increase of α-MSH in CFS patients, and we could not rule out a possibility that this was simply an empirical association.

Currently an intriguing issue would be an association between viral infections and α-MSH levels. Recently, a link between the xenotropic murine leukemia virus-related virus (XMRV) and CFS was reported and suggested that XMRV infection may be a causal factor in the pathogenesis of CFS [25,26]. Although this association is currently conflicting and under debate, changes of inflammatory cytokines and chemokines by XMRV infection may lead to the increase of α-MSH levels. It would be of interest to clarify the association between XMRV infection and increase of α-MSH levels.

Although α-MSH could be a biomarker for CFS within five years duration, the α-MSH level may be higher in other fatigue related diseases such as insomnia, sleep apnea and inflammatory diseases caused by virus infections. Those points should be clarified in successive studies near future.

## Conclusions

In conclusion, increased plasma levels of α-MSH are found in patients with CFS during the first 5 years of the disease. Although raised α-MSH levels are also observed in CHF, obesity, and inflammatory diseases such as sepsis and HIV, all these diseases can be diagnosed and excluded as diagnoses in patients with CFS. After exclusion of these other diseases, α-MSH has the potential to act as a biomarker for CFS. Further studies of fatigue-related diseases are needed to confirm its potential and establish the reliability of α-MSH as a marker of CFS.

## Abbreviations

CFS: Chronic fatigue syndrome; α-MSH: alpha-melanocyte stimulating hormone; ACTH: adrenocorticotropic hormone; DHEA-S: dehydroepiandrosterone sulfate; RIA: radio immuno assay; BMI: body mass index; LPS: lipopolysaccharide; HIV: human immunodeficiency virus; TNF-α: tumor necrosis factor alpha; CHF: congestive heart failure.

## Competing interests

The authors declare that they have no competing interests.

## Authors' contributions

NS-I carried out the radio-immunoassay (RIA) for α-MSH and the statistical analysis, and drafted the manuscript. TO participated in the design of the study and the RIA. KY and HKu diagnosed CFS patients, collected blood samples and provided other information for CFS patients. YW and HKu participated in the design of the study and interpretation of the data and drafted the manuscript. HKi conceived of the study, participated in its design and coordination and drafted the manuscript. All authors read and approved the final manuscript.

## Pre-publication history

The pre-publication history for this paper can be accessed here:

http://www.biomedcentral.com/1471-2377/10/73/prepub
